# Perception of AI Use in Youth Mental Health Services: Qualitative Study

**DOI:** 10.2196/69449

**Published:** 2025-08-19

**Authors:** Xiaoxu Ding, Skye Barbic

**Affiliations:** 1 Department of Occupational Science & Occupational Therapy, Faculty of Medicine, University of British Columbia, 317-2194 Health Sciences Mall, Vancouver, BC, V6T 1Z3, Canada, 1 2369928222; 2 Foundry, Vancouver, BC, Canada

**Keywords:** artificial intelligence for health, youth mental health, healthcare service delivery, digital health intervention, stakeholder perception

## Abstract

**Background:**

Artificial intelligence (AI) technology has made significant advancements in health care. A key application of using artificial intelligence for health (AIH) is the use of AI-powered chatbots; however, empirical evidence on their effectiveness and feasibility remains limited.

**Objective:**

This study explored interest group perceptions of integrating AIH in youth mental health services, focusing on its potential benefits, challenges, usefulness, and regulatory implications.

**Methods:**

This qualitative study used semistructured in-depth interviews with 23 mobile health stakeholders, including youth users, service providers, and nonclinical staff from an integrated youths’ service network. We used an inductive approach and thematic analysis to identify and summarize common themes and subthemes.

**Results:**

Participants identified AIH’s potential to support education, navigation, and administrative tasks in health care, as well as to create safe spaces and mitigate health resource burdens. However, they expressed concerns about the lack of human elements, such as empathy and clinical judgment. Key challenges included privacy issues, unknown risks from rapid technological advancements, and insufficient crisis management for sensitive mental health cases. Participants viewed AIH’s ability to mimic human behavior as a critical quality standard and emphasized the need for a robust evaluation framework combining objective metrics with subjective insights.

**Conclusions:**

While AIH has the potential to improve health care access and experience, it cannot address all mental health challenges and may exacerbate existing issues. While AIH could complement less-complex services, it could not replace the therapeutic value of human interaction at this time. Co-design with end users is critical for successful AI integration. Robust evaluation frameworks and an iterative approach to build a learning health system are essential to refine AIH and ensure it aligns with real-world evolving needs.

## Introduction

Over the last decade, artificial intelligence (AI) has made significant breakthroughs in health care [1]. More advanced AI technologies, such as machine learning [2], natural language processing [3], and predictive analytics [4], have increasingly been introduced to diverse health care settings to support diagnostic capabilities, individualized treatment planning, administrative and clinical workflow development, and patient monitoring [5]. Using artificial intelligence for health (AIH), especially in the field of youth mental health, is in an exploratory phase. The current youth mental health landscape is often critiqued as fragmented and insufficient to meet the access and care needs of diverse youths [6,7]. AI offers a promising solution to augment existing services, with its low barriers to entry and resource-efficient nature, capable of enhancing existing services by providing real-time, data-driven support [8]. Recent advancements in generative AI, including large language models, further extend these possibilities by offering capabilities such as real-time emotional recognition, therapy-session summarization, crisis risk prediction, and personalized psychoeducation [9].

Given that youths (defined here as 12–24 years) are generally more receptive to new technologies than other age groups [10], they are uniquely positioned to lead the adoption of AI-based mental health services. The integration of AI into these services not only has the potential to revolutionize care delivery but also to improve health outcomes and experiences, promote population health, reduce costs, and enhance both provider satisfaction and health equity, aligning with the goals of the quintuple aim [11]. However, these developments also introduce significant challenges, especially given the sensitive nature of mental health data and the critical importance of human empathy and therapeutic relationships in youths’ care [9,12]. Therefore, the integration of AI into youth mental health services must carefully consider issues such as algorithmic bias, transparency, value alignment, and the potential loss of humanistic care elements.

Despite the challenges stemming from rapid advancements in this technology, there is a significant gap in evidence on how these AI innovations translate into successful AIH implementations. Perceptions of AI in health care remain mixed [8], especially in areas where AI is more embedded in digital health interventions, remote monitoring, and preventive care [13-15]. Stakeholders such as youth users, health care providers, technology developers, and policy makers hold pivotal roles in shaping the acceptance, regulation, and application of these technologies [16]. Their perspectives are critical in ensuring AIH solutions are tailored to the real-world needs of youth mental health services, rather than just performing in idealized experimental settings. This gap highlights the urgent need to engage with these stakeholders, whose insights are essential for fully understanding both the potential and the limitations of AI in transforming youth mental health care.

This paper explores the perceptions of key interest groups on the integration of AIH into youth mental health services. Specifically, we examine the (1) benefits and challenges of AIH integration, (2) perceived usefulness of AIH, and (3) strategies for evaluating and regulating AIH. By addressing these critical questions, this study sheds light on the factors influencing AI adoption in mental health care and offers actionable recommendations to support the responsible, equitable use of AI to improve care quality and accessibility for youths.

## Methods

### Study Design

This study used an inductive qualitative approach with semistructured, in-depth interviews to explore stakeholder perceptions of integrating AI-based tools (AIH) into youth mental health services. This study was situated within Foundry, a provincial network of integrated youth services (IYS) in British Columbia, Canada. Foundry offers youths aged 12–24 years access to mental health and substance use services, primary care, social services, and peer support. Foundry operates both physical centers and a virtual mHealth (mobile health) platform (the Foundry BC [British Columbia] app). Although Foundry does not currently offer AIH, its active digital infrastructure and dedicated mHealth team make it a potential setting for exploring the potential of future AI integration.

Three priority participant groups were included: (1) youth users, (2) service providers, and (3) nonclinical staff. These groups were selected to capture diverse perspectives across different stages of technology design, development, and implementation. Youths and service providers represent the primary users and deliverers of mHealth services, while nonclinical staff offer critical insights into the operationalization and governance of online health tools.

### Ethical Considerations

Ethical approval was received from the University of British Columbia Office of Research Ethics Behavioural Research Ethics Board (#H22-03454). Study findings are reported in alignment with the COREQ (Consolidated Criteria for Reporting Qualitative Research) checklist for qualitative studies. Verbal and written consent was obtained from all participants prior to the interviews. Interviews lasted between 45 and 60 minutes, were audio-recorded with consent, and transcribed verbatim. Field notes were taken to aid data cleaning, capture nonverbal observations, and assist in contextual interpretation. Each participant was coded with a pseudonym for confidentiality purposes and anonymous presentation of results. Participants received a CAD $50 (US $36.30) honorarium after their session.

### Study Sample

All recruitment and data collection took place between June 2023 and April 2024.

#### Youth Users

Youth inclusion criteria required participants to be aged 16 to 24 years (those 15 years and younger were excluded due to the need for parental consent), able to communicate in English, and have used mHealth to access services in the past year. Recruitment was conducted through recurring social media posts. To capture diverse experiences, no restrictions were placed on the frequency or purpose of mHealth use.

#### Service Providers

For this group, we recruited IYS service providers (eg, counselors, social workers, primary care providers) who have used mHealth to deliver care to youths (eg, virtual youths’ counseling, remote info sessions, and online peer support groups). Most service providers were purposively recruited from Foundry centers that fully integrate mHealth into their clinical service workflows.

#### Nonclinical Staff

For this group, we recruited technology and implementation experts at Foundry who were engaged in the design, development, and implementation stages of the mHealth platform. We reached out to the Foundry communications team to share information about this study’s opportunity to qualified nonclinical mHealth staff who met the inclusion criteria. This process was used to ensure the confidentiality of staff so they could make an unbiased decision to participate in the interviews.

### Data Collection

We collected qualitative data through 23 participant interviews. We designed open-ended questions based on the participants’ own perception and user experience with AI and AIH. To ensure a shared foundation for discussion, each interview began with a brief conversation about AI and AIH, helping to ensure that participants’ understanding aligned with commonly accepted definitions of these concepts. The major guiding questions were constructed based on the Technology Acceptance Model (TAM) [17-19]. TAM’s core constructs informed the formulation of questions aimed at understanding participants’ views on the potential integration of AI into youth mental health services. Interview questions probed stakeholders’ beliefs about how AI could improve health care services (usefulness), their concerns about complexity or usability (ease of use). Specifically, TAM3 was taken into consideration because it further integrates broader factors such as trust, relevance, and ethical considerations that could influence acceptance in a youth mental health context. The semistructured interview format allowed participants to elaborate on topics of interest beyond the guiding questions (sample interview questions listed in Multimedia Appendix 1). Examples were offered when necessary (eg, “imagine using a chatbot to ask questions about anxiety or depression or to help you book a mental health appointment”). This framing helped participants consider the role of AI beyond general uses and reflect on potential health care applications, even if they had not personally used AI for mental health purposes.

Interviews were conducted via secure Zoom by the lead researchers (XD and SB) with qualitative research training and prior experience in the mHealth service setting.

### Data Analysis

We used inductive thematic analysis [20,21] to identify, analyze, and report patterns and themes in interview data from 3 stakeholder groups [22]. Researchers XD and SB used an iterative approach to review the themes, ensuring they accurately represented the coded data and the overall dataset content. After identifying themes for each research question (RQ), the authors discussed and selected the most representative examples from the transcripts for each theme, presenting in-depth quotes alongside the group name and a pseudonym for each participant. To ensure rigor [23], the research team held weekly debriefs to review theme development and discuss discrepancies in interpretation. Reflexivity was maintained through memo writing and regular team reflection, particularly around the influence of positionality in interpreting different stakeholder perspectives. Themes were finalized once data saturation was achieved and no new codes emerged from subsequent transcripts.

## Results

### Participants

A total of 23 people participated in this study, with 12 youth users, 6 service providers, and 5 nonclinical staff who were deeply involved in the development of the mHealth services across the IYS network. Table 1 summarizes the demographic characteristics reported by the participants. Most participants self-reported using AI-powered tools in their daily lives, not limited to health contexts. Many had used AI tools, most commonly ChatGPT (OpenAI), for tasks related to school, work, and everyday problem-solving. Two youths reported they had used Snapchat (Snap Inc) for AIH-related counseling purposes. Most participants provided diverse and distinct insights on their perception of AIH IYSs, ranging from “it’s scary and creepy” and “I am skeptical” to “it has potential” and “it is a positive trend.” Only 1 participant in the youths group stated that they had never thought about using an AIH-related service and did not provide much information.

Through one-on-one interviews, participants shared their in-depth understanding of the current and future role of AI, specifically the integration of generative chatbot services in health care, based on their personal experiences. Thematically analyzed qualitative data will be presented in this Results section following the 3 research questions proposed: perceived benefits and challenges of AIH, intended usefulness of AIH, and how do we evaluate AI for regulation.

**Table 1.** Summary of demographic description of 3 groups (N=23).

	Youth (n=12)	Service providers (n=6)	Nonclinical staff (n=5)
Age (years), mean (range)	20.4 (18‐24)	32.8 (23‐45)	35.6 (29‐46)
Self-reported gender, n			
Woman	10	4	3
Man	2	1	1
Nonbinary	0	1	1
Currently using AI in life, n (%)	11 (92)	5 (83)	4 (80)
Years of professional experience, mean (range)	N/A	4.2 (1.5-7)	14.1 (8‐23.5)

### RQ1: Perceived benefits and challenges of AIH

#### Perceived Benefits of AIH

##### Create a Safe Environment

When youths accessed virtual care, there were unique preferences for everyone. Some expected a real person on the other end of the screen, and some youths reported they have a strong fear of judgment, stigma, and social anxiety when facing a therapist. These participants reported that the lack of human interaction is beneficial in their help-seeking journey. This consideration can be particularly crucial in vulnerable groups, as the LGBTQIA (lesbian, gay, bisexual, transgender, queer, intersex, and asexual) community reported facing additional barriers when accessing mental health services.

*The wall is my own struggling to trust. It’s just my own wall that I don’t want anybody to know what I’m struggling with. Because, like you say, it’s an AI, so it’s not a person who will know my struggle.*

[Youth, July]

*It feels wrong to suddenly question their attraction to the same sex, and I had a young person said that to me, ‘I’m so embarrassed I could never tell my friends, I could never tell my parents, I could never tell anyone but I needed to tell somebody.*

[Nonclinical staff, Sarah]

##### Mitigate Health Resources Burden

Participants reported that AIH is naturally perceived as affordable, resourceful, and available 24/7. It can provide immediate responses they need without having to go through a complicated registration and waiting process, compared to how you usually access a traditional therapy session. Some service providers optimistically suggested that AI could easily replicate certain therapeutic approaches that are relatively straightforward, such as the solution-focused brief therapy model and attention-deficit/hyperactivity disorder coaching. They proposed that AI could be designed to deliver appropriate responses at the right time. Participants suggested that if AIH can effectively and accurately handle less complex cases, it could alleviate the current shortage of health care resources. This would enable the system to dedicate more focus and resources to addressing higher-intensity situations.

*Our clinical staff who worked at a help line reported that mostly people just want to talk to somebody, and who just feel like they need maybe some guidance or someone to listen to them, and this is the part that can benefit from a well-trained AI model.*

[Service provider, Milo]

### Perceived Challenges of AIH

#### Missing Human Element

As all participants have their AI experiences with generative AI chatbots, they largely envision using ChatGPT-like chatbots for therapeutic purposes. While the technology team showed confidence that AI has the capability of feeding the correct answer, most clinical staff and youths suggested that the value of talking to service providers is building empathetic relationships and connecting with the community. Current AIH cannot understand client facial cues, tones, raised voices, or body language, and to provide human-like empathetic responses (“You know they are crying, AI doesn’t” [Nonclinical staff, Allison]). This perspective is particularly crucial when it comes to trauma counseling, crisis counseling, and suicide cases, since most participants stated AIH cannot handle extreme situations that require extra considerations and empathy. Moreover, both youths and service providers shared from their counseling experiences that clients often come in feeling vulnerable and seek to share that sense of vulnerability with another human being present in the same space. Sometimes clients are not here to hear the right words; they are here to feel heard and supported—“But you are not able to feel that from any robots” (Youth, John).

*Your counselor is a human, they have human emotions, they make mistakes, they say weird things, too, and it’s very reassuring to know that the person we’re speaking with, despite being a professional counselor, they’re also just living the human experience. Even if a counselor says the wrong thing, they were cursing with you that AI will never do, but you still know that they are there to support you.*

[Youth, Rice]

In addition to the lack of empathy, service providers also reported that AIH lacks the clinical judgment ability and the power of uniqueness, so it is not personalized at all when facing different clients.

*Two people could have the same issue. But then with an AI, if it’s given the same prompt, it would probably have the same answer for both. But I feel like human therapist can change it up per person or being able to read the conversation better and make inferences during session.*

[Youth, Sisi]

#### Worries About Technology

Participants from all 3 groups expressed concerns about the feasibility of implementing AIH services, particularly regarding the ability of health services to meet technological demands and address the evolving needs of youths. Participants also discussed equitable access to technology. Some participants noted that it is crucial to recognize there are rural and remote communities that do not have access to technology (eg, Wi-Fi), and some were not comfortable accessing the internet and remote services. While the AIH has a promising future, participants acknowledge that it is not the solution for every community, and the needs of each community need to be carefully scoped. As 1 participant noted,

*It could be more harmful than good to do that [implement AIH] in communities where it’s does not really aligned with how they live.*

[Nonclinical staff, Lulu]

Participants also identified confidentiality and privacy as key concerns regarding the logistics of AIH. While these issues are common in all technology-based services, participants noted that they are particularly challenging in AIH because users often lack a clear understanding of who or what is managing the information they input into the “black box.”

*Will that be private, or will it go through some counselors I don’t know or just to Google?*

[Youth, July]

Some participants also perceived that AI in general can lack effectiveness and reliability, which is critical for delivering evidence-based services to youths accessing mental health services. Some perceived AIH as “impractical,” and some participants reported highly negative experiences with AI chatbots, leading to a strong reluctance to see AI integrated into their health care experiences.

*It’s frustrating enough dealing with something as simple as Amazon customer service, let alone relying on AI for health-related matters. Anytime I can tell I’m talking to a robot, my first thing is to figure out how to get to the human.*

[Nonclinical staff, Allison]

#### Risks

In addition to the general concerns toward using technology, participants also proposed more serious risks associated with integrating AIH into the health care system. Some stakeholders believed that at this stage, “AIH has more risks than what current knowledge can anticipate” (Service provider, Jacob).

*I don't think people like the idea of getting therapy from a program.*

[Service provider, Olivia]

Participants noted that unregulated AI tools can be maliciously trained, spread misinformation, and, more critically, lack empirical research evidence on the negative consequences resulting from such misconduct. All participant groups emphasized that each user interacts with AIH in unique ways, making it difficult to predict the specific information these tools provide.

It is important to note that nearly all participants expressed concerns about how difficult it can be to manage crises with AIH. This was identified as the most significant worry and the primary challenge when integrating AIH into youth mental health services. Participants specifically stressed the importance of exercising extreme caution with AI tools, highlighting the risk of these tools delivering triggering or harmful content that could lead to self-harm or suicide.

*I worked with a couple of projects that was using AI to train particular counseling or training models. Right now I’m suspicious because you can make AI mad at you. I remember this…not ChatGPT, but a while ago I managed to convince the AI to tell me to kill myself and sent that back to somebody and …this is a no.*

[Nonclinical staff, Allison]

Participants expressed a desire for AIH to be accompanied by a comprehensive crisis management plan that addresses the handling of sensitive information while prioritizing ethical and legal considerations. Finding a balance between data security and effective crisis management was described as a significant challenge for all groups.

*To do a suicide rescue with somebody on AI is intense. Do you need to check for other things like do they have the modality? Do they have a plan? Is the plan imminent? So where is the line to necessarily get other people involved? If it looks like they’re at a high risk for suicide, at that point somebody would need to know? But also there are also health laws there, somebody else would never be able to involve.*

[Service provider, Flora]

### RQ2: Current Intended Usefulness of AIH

Participants expressed 3 key functions where AIH could play to advance youth mental health service innovation.

#### Education

First, based on participant experiences with AI tools, participants felt that AI can serve as an effective educational resource to support the learning of health-related knowledge. AI can answer scientific questions without waiting times (eg, “What is an antidepressant?”) and can provide tailored materials for diverse audiences, such as explaining medical concepts to youths in plain, accessible language. Additionally, it can update both health care providers and recipients with the latest knowledge and skills that are personalized to their specific needs. AI can help foster a more informed and knowledgeable support system and bridge gaps in health literacy.

*If you’re in need of realistic advice that you don’t really need an appointment for, maybe AI can help. If I can get the solution right away. then [using AI to seek help] wouldn’t be a concern for me.*

[Youth, Kate]

#### Navigation

Second, participants reported that AI can be a navigation tool that directs users to the correct place to seek help. Participants suggested that AI chatbots can be used as screening tools to assist with identifying the type of support they need based on their symptoms or concerns and direct them to the suitable health care providers, facilities, or online resources. Participants proposed that AI can be trained with the stepped care model [24] and help triage users in specific communities by recommending whether they should seek immediate emergency care, schedule an appointment with a specialist, or explore self-management options. By navigating users across the complex health care system, AI has the potential to increase access to care, minimize delays, and mitigate stress for individuals in need of accessible services, especially those from vulnerable and marginalized groups, including youths.

*I can definitely see to use it like find me a center near me,* “*okay, you have one x kilometers away,” or* “*here is a substance use support station for you” and it would be cool. But I’m very against the idea of AI being my counsellor.*

[Youth, Rachel]

#### Administration

Lastly, participants agreed that other than using AI for accessing health care, AIH has extensive value for assistive health administration purposes. Many youth users, especially those whose first language is not English, suggested that AI services can help overcome language barriers by accurately expressing their thoughts in their native language, often performing better than traditional translation tools. Service providers also highlighted that AI can handle administrative tasks such as appointment scheduling, billing, and managing patient records. This reduces the workload for health care staff, allowing them to focus more on providing personalized care. Additionally, AI can analyze health data to identify patterns in service use, track both short-term and long-term patient records, and support decision-making at the organizational level.

*You can have AI store all the data and generate tables for like… what percentage of people accessed the app this month, and you will know the maintenance and other tech efforts you will need in the future.*

[Nonclinical staff, Allison]

### RQ3: Evaluation and Regulation of AIH in Youth Mental Health

All participants highlighted the importance of assessing the quality of care delivered by AIH and identifying effective regulatory measures to maximize its benefits for youths’ mental health. At the beginning of this section, it is important to highlight that the prevailing view among stakeholders is that the success of AI-based health care services largely depends on how well AI can mimic human behavior. Many emphasized that AIH should incorporate human-like traits, especially empathy, to build trust and gain acceptance. The importance of having diverse personalities in AI was repeatedly emphasized by different participants, with some suggesting that users should be able to choose the different personalities of AI based on the specific service they are using. Stakeholders agreed that aligning AI with these desired qualities is key to its effective integration into youths’ mental health care.

*I would want to see whatever I can see in a real person, then it would actually be the same thing. If they didn’t have this, then I wouldn’t be satisfied. I want AI to be an active listener, so should be empathy! I want the AI chat to have empathy. I want it to be non-judgmental. I want the chatbot to challenge me in my thoughts and my patterns like a real therapist.*

[Nonclinical staff, Alex]

Building on the overarching standard proposed by participants that AI services should mimic human behavior, 2 major categories of evaluation criteria were identified: objective measures based on quantifiable metrics, and subjective assessments based on user experiences.

The quality of care provided by AIH can be objectively assessed by tracking changes in symptom severity, using tools such as the GAD-7 (Generalized Anxiety Disorder 7-Item) and PHQ-9 (Patient Health Questionnaire-9 Item) scales to measure anxiety and depression levels in youths before and after the intervention. In addition to symptom severity, participants suggested other measurable factors that could be part of a comprehensive evaluation framework. These include the percentage of accurate information provided, response times, frequency of follow-up interactions, the number of successful referrals to appropriate resources, and even the reduction in years of disease burden at the population level.

*Is it cutting down on the number of people who then go on to book an appointment? How effective it is in achieving individual health goals? Did it convince youth to take the next step to see a specialist? You can calculate some efficiency percentage here.*

[Nonclinical staff, Jojo]

The other perspective is that you can measure the subjective individual user experience and level of satisfaction while using AIH. Participants noted that lived experiences are difficult to quantify and should not be categorized, as they often provide the best reflection of the unique perspectives, emotions, and challenges individuals encounter, shaped by their personal and cultural backgrounds. This part of the evaluation can include highly subjective feedback, such as: “Did I feel heard and understood? Did I receive the response I needed from this chat session? Did I feel empathy and validation? Did I feel safe talking to AI? Did I feel supported? Was the level of service consistent across sessions? Was I able to reach out to the kind of service I need?”

*When I was typing something on Snapchat, and then it gives me something back… like a huge paragraph, and I read over, and I’m like, OH, you just completely got it in a wrong way. So I don’t even have the energy to continue and to write to AI ‘you’re wrong’. So for me it did not give like a really good response and it was a waste of my time.* [Youth, Sunny]

## Discussion

### Principal Findings

The integration of AI into youth mental health services presents both opportunities and challenges. This study explored the perceptions of mHealth interest groups who are already familiar with mHealth services, offering critical insights into the benefits and challenges associated with integrating AIH in a real-world setting. Participants expressed the expectation that AIH could enhance care by improving health education, service navigation, and supporting administrative tasks. At the same time, participants proposed concerns about the loss of human empathy, lack of clinical judgment, data privacy risks, and the inability of AI to handle high-risk situations such as mental health crises. These findings emphasized the need for thoughtful AIH implementation that is more tailored to unique needs.

Previous evidence has highlighted the potential of using AI-powered health tools to address key barriers in health care, such as workforce shortages and financial constraints, by offering data-driven mental health interventions [1,25]. Some research has explored the use of AI in clinical decision-making, such as optimizing drug dosages and creating personalized treatment plans [26,27]. However, regarding implementing AIH to support mental health services, the American Psychiatric Association’s *DSM* (*Diagnostic and Statistical Manual of Mental Disorders*) includes over 450 distinct definitions [28] of mental disorders, and current research does not have empirical evidence to support the use of AIH in all fields of mental health services.

In addition to these concerns, advancements in health technology often fail to engage end users effectively and neglect their lived experiences and needs [16,26]. While existing evidence showcases the capabilities of AIH, there is limited exploration of how service recipients perceive its use in a practical setting. This study investigated stakeholder perceptions, emphasizing the role of AIH, particularly AI chatbots, in supplementing traditional services. A key challenge identified was AI’s inability to replicate human empathy, which aligns with some scholarly views [29], and this is especially crucial in critical situations requiring nuanced therapeutic responses. Recently, more research has focused on understanding the warmth and empathy conveyed by chatbots. Some showed empathy expressed by a chatbot may feel inauthentic [30], and users often prefer human-written stories over those generated by AI in mental health and social support settings [31]. Others, however, see potential in enhancing AI chat features and making them more empathetic and responsive to patient experiences [32]. This reflects the ongoing scholarly debate around the topic of AI and empathy, leading to a contentious aspect of AIH integration.

Another recurring concern identified by participants in this study was the fear of AI mishandling sensitive data and spreading misinformation, particularly in high-risk situations for youths. Existing studies identified both technical and ethical risks associated with AIH, including the spread of misinformation about mental illness that contains factual errors, misleading claims, invented references, or advice that may be unsafe in crisis management and clinical contexts [33,34]. Literature underscores the importance of service providers acknowledging this risk and developing adaptive strategies for practice [33]. Some researchers have proposed using a “supervisor AI” to identify and correct misinformation, particularly on social media, but the feasibility of integrating such systems into AIH remains uncertain [35,36]. The study highlighted the need to expand evaluation criteria for AIH. While traditional measures, such as symptom reduction, remain important, there is increasing recognition of the complexity involved in measuring AIH tools [37,38]. Participants argued that a more holistic approach is necessary, focusing on evaluating meaningful, subjective recovery experiences, rather than solely relying on quantitative metrics. Lastly, participants emphasized the importance of efficiency and brevity in AIH interactions. Youths described disengaging after receiving lengthy and misaligned responses. This reflects a long-lasting usability issue in digital platforms [39,40], where users may feel that their time is “disrespected.” As we are discussing AIH integration in youth mental health settings, it is essential to tailor responses to youths’ cognitive load to sustain engagement and therapeutic value.

### Limitations

For this qualitative study, the interview data came from a small sample within one youth service network, limiting the generalizability to broader contexts. Participants primarily shared perceptions of AIH integration based on their personal AI experiences, as they had limited direct experience with implemented AI-based health tools in a clinical youth mental health setting, which may have limited the depth of their insights.

### Future Endeavors

Beyond the potential functions of AIH identified by participants, its effectiveness in health care can be enhanced by strategically integrating AIH applications with established care models, such as the stepped care model. This approach may allow AI to manage lower-complexity cases, enabling clinicians to focus on high-intensity, complex cases in youth mental health, thereby improving overall treatment outcomes. Moreover, to build trust and encourage widespread adoption in youth mental health, AIH must prioritize transparency, especially regarding data management and crisis intervention. Establishing robust ethical guidelines and regulatory frameworks is crucial to ensuring AI safety and addressing any potential risks. Most importantly, even as the technology matures, AIH solutions must be co-designed with end users, ensuring they are tailored to meet their needs and foster trust in the health care system. Given the current limitations of AIH integration reported by participants, there is a need for health care systems to adapt iteratively to the evolving needs of users, especially when it comes to vulnerable groups such as youths who usually face more barriers and challenges when accessing care. The future development of AIH should also prioritize continuous feedback and foster collaborative learning environments involving all interest groups. This includes groups represented in this study, as well as others not recruited, such as organizational leaders and policy makers. Our sample was small and predominantly composed of women who were relatively tech-savvy with mHealth tools but had limited direct experience with AI-powered health tools used in a clinical context. Broader representation across gender, background, and AI experiences may yield additional insights and ensure findings are more representative and actionable. While participants’ perceptions offer valuable direction for early-stage design, future research should include more diverse and experienced stakeholders to inform equitable and tailored AIH development. This effort aligns with the call for a learning health system [41] that supports long-term interest groups’ engagement rather than isolated, project-based approaches to break down silos among partners and to foster collaboration across AIH design, development, and implementation stages. Finally, it is crucial to recognize that while participants in this study mainly believed the current health care system is not yet prepared to fully integrate AI services, these perceptions are likely to evolve as technology and system development progress. As such, the establishment of a learning health system could provide the ongoing feedback and continuous improvement required to effectively integrate AIH, ensuring its adaptation and growth in alignment with the needs of youth mental health providers, service users, and technology developers.

### Conclusion

This study underscores both the promising potential and significant challenges of integrating AI into youth mental health services. AI tools can be used for education, navigation, and administrative purposes. AIH can help create accessible environments and alleviate the burden on health care resources, yet its limitations cannot be overlooked. These include the unknown risks associated with current AI technology, the absence of essential human elements in care, the lack of effective crisis management plans, and the absence of a comprehensive regulatory framework for its integration into mental health systems. Additionally, there is a pressing need to develop a robust evaluation framework and establish ethical oversight to ensure AIH can adapt to the evolving needs of youth mental health services. Moving forward, it is critical to focus on building a learning health system for continuous improvement that encourages collaboration, ensuring AIH solutions are effective, equitable, and sustainable for future generations.

## Supplementary material

10.2196/69449 Multimedia Appendix 1 Sample interview questions.

## Abbreviations

AI: artificial intelligence
AIH: artificial intelligence for health
COREQ: Consolidated Criteria for Reporting Qualitative Research
*DSM*: *Diagnostic and Statistical Manual of Mental Disorders*
GAD-7: Generalized Anxiety Disorder 7-Item
IYS: integrated youths' service
LGBTQIA: lesbian, gay, bisexual, transgender, queer, intersex, and asexual
mHealth: mobile health
PHQ-9: Patient Health Questionnaire-9 item
RQ: research question
TAM: Technology Acceptance Model
