# Supplementary material for: Perception of AI Use in Youth Mental Health Services: Qualitative Study
Source: J Particip Med. 2025 Aug 19;17:e69449. doi: 10.2196/69449 (PMC12364429; doi:10.2196/69449)
Supplement: Multimedia Appendix 1 [file jopm-v17-e69449-s001.docx]

1. How is AI being used in your daily life? Both health related and not health related.

2. How do you see its usage in the healthcare system?

3. What about its implication to the current mHealth services?

4. What do you think AI should be used for? Particularly in health?

5. If you're the Minister of Health, and you can use all the resources. How would you like to use it for AI health tools?

6. What do you think is the current challenging part of implementing AIH?

7. So you were talking about asking questions on ChatGPT, and if the current Foundry service is transferred into AI chat-based counseling, how would you react? Would you still consider using it?

8. Your answer, was it due to trusting the information?

9. So what if people actually put AIH into daily uses, what are some aspects that you believe should be valued the most in AI service?
